# A GPU-accelerated algorithm for biclustering analysis and detection of condition-dependent coexpression network modules

**DOI:** 10.1038/s41598-017-04070-4

**Published:** 2017-06-23

**Authors:** Anindya Bhattacharya, Yan Cui

**Affiliations:** 1 Department of Microbiology, Immunology and Biochemistry, Memphis, TN 38163 USA; 2 Center for Integrative and Translational Genomics, University of Tennessee Health Science Center, Memphis, TN 38163 USA; 3 Department of Computer Science and Engineering, University of California, San Diego, CA 92093 USA

## Abstract

In the analysis of large-scale gene expression data, it is important to identify groups of genes with common expression patterns under certain conditions. Many biclustering algorithms have been developed to address this problem. However, comprehensive discovery of functionally coherent biclusters from large datasets remains a challenging problem. Here we propose a GPU-accelerated biclustering algorithm, based on searching for the largest Condition-dependent Correlation Subgroups (CCS) for each gene in the gene expression dataset. We compared CCS with thirteen widely used biclustering algorithms. CCS consistently outperformed all the thirteen biclustering algorithms on both synthetic and real gene expression datasets. As a correlation-based biclustering method, CCS can also be used to find condition-dependent coexpression network modules. We implemented the CCS algorithm using C and implemented the parallelized CCS algorithm using CUDA C for GPU computing. The source code of CCS is available from https://github.com/abhatta3/Condition-dependent-Correlation-Subgroups-CCS.

## Introduction

Clustering algorithms have been widely used to group genes based on their similarities in expression^1–4^. The gene expression coherence is often related to functional coherence. A recent comparative assessment of 21 existing clustering algorithms showed that the clustering algorithms report more functionally meaningful clusters by exploiting the relationships between all pairs of genes^1^. Clustering algorithms are also known to perform better grouping of co-functional genes if they search for similarities between expression patterns rather than similarities between expression values^1, 5^.

Functional relations between genes may vary over conditions^6, 7^. For example, a group of genes may act coherently under one set of conditions but may become inactive or perform different functions separately under another set of conditions. Clustering algorithms that obtain grouping based on similarities over all the samples in a dataset are not effective for detecting condition-dependent coexpression patterns. Biclustering algorithms have been developed to address this problem^1, 8–12^. A bicluster consists of a group of genes and a set of conditions under which the genes are co-expressed. Searching for bicluster is a challenging problem because the number of potential biclusters is exponential to the number of genes and samples. An important difference between various biclustering algorithms is how they apply heuristic rules to detect biclusters. Most biclustering algorithms use local search heuristics that may miss many biclusters. Conventional way of finding biclusters depends on the selection of random seeds of genes and/or samples followed by their augmentation based on a scoring function. However, random selection of initial seeds is often unable to efficiently cover the search space and to obtain a consistent set of biclusters from multiple runs on the same input data. The selection of scoring function for the heuristic search is also important for finding the biologically meaningful biclusters. Most common biclustering approaches adopt arithmetic mean of the gene expression or an up/down-regulation patterns on corresponding discretized data matrix. Both are inefficient for finding the interesting co-regulatory modules where genes are expressed with similar or opposite patterns of expression but the expression values are very different. Such modules are important as they may represent relations between genes in the same biological functions^1, 13^.

A Pearson Correlation Coefficient based scoring function was introduced by Correlated Pattern Biclusters (CPB)^14^. However, the performance CPB is still depends on the random selection of samples. Benefits of Pearson Correlation Coefficient based similarity measures over the conventional mean square residue based bicluster scores again was demonstrated by Bi-Correlation Clustering algorithm (BCCA) that looks for positively correlated biclusters and reports biclusters for each pair of genes present in a dataset^13^. Initially, for each pair of genes all the samples are selected and then a greedy search is made to eliminate samples one at a time until the gene pair shows positive correlation higher than a predefined high positive correlation threshold. The gene pair is then augmented with other genes that are correlated to form a bicluster. However the backward elimination of samples in a greedy search is subject to finding a local optimal subset of samples, hence in reality it imposes a restriction on the search space for finding the optimal set of biclusters. BIclustering by Correlated and Large number of Individual Clustered seeds (BICLIC) introduces another alternative correlation based biclustering. BICLIC forms biclusters by starting from a large number of clustered seeds followed by augmentation and deletion of rows and columns based on the correlations with seeds^15^. The main drawback of BICLIC which is also true for BCCA is finding a large number of overlapping biclusters which are very much identical. Moreover, BICLIC, BCCA and other correlation based biclustering methods, only look for finding positively correlated gene groups as bicluster. However, in reality negatively correlated genes are equally relevant and may represent important regulatory mechanisms in biological functions.

Here we propose a more effective correlation-based biclustering algorithm named Condition-dependent Correlation Subgroup (CCS). It integrates several important features for developing an effective algorithm for comprehensive discovery of functionally coherent biclusters^1^. A significant challenge in genomic data analysis is to utilize the fast growing high performance computing capacity to process and analyze large complex datasets efficiently^16–18^. CCS is particularly suitable for parallel computing. We used the GPGPU computing for a parallel implementation of the algorithm in CUDA C which shows a substantial speedup compared to the sequential C program. The performance of CCS was compared with CPB, BCCA, BICLIC and ten other widely-used biclustering algorithms on 5 synthetic and 5 real gene expression datasets. CCS outperforms the other biclustering algorithms in all the comparisons. We also showed that there is equivalence between the CCS biclusters and condition-dependent coexpression network modules.

## Methods

A bicluster is a group of genes with a related pattern of expression over a group of samples. We defined the related pattern of gene expression in terms of positive and negative Pearson correlation coefficients. Let us consider a gene expression data set D = G × E where G = {g_1_, g_2_, …, g_n_} is a set of “n” genes and E = {e_1_, e_2_, …, e_s_, …, e_m_} is a set of “m” samples. For each gene g_i_ there is an m-dimensional vector x_i_. In vector x_i_, x_is_ is the value of e_s_ for gene g_i_. In our algorithm we defined a bicluster as a group of genes “I” over a group of samples “J” where each gene g_i_ in “I” is correlated to all the other genes g_j_ in “I” with an absolute correlation value greater than a threshold θ. Mathematically a bicluster “C” is represented as C = (I; J) where “I” is a subset of “G” and “J” is a subset of “E”. The Pearson correlation coefficient between g_i_ and g_j_ over a subset of samples “J” is represented as r(g_i_, g_j_)_J_ and defined as

1 $$r{(gi,gj)}_{J}=\frac{{\sum }_{s=1}^{m}(({x}_{is}\times {b}_{s}-\bar{{x}_{i}\times b})({x}_{js}\times {b}_{s}-\bar{{x}_{j}\times b}))}{({\sum }_{s=1}^{m}{({x}_{is}\times {b}_{s}-\bar{{x}_{i}\times b})}^{2})({\sum }_{s=1}^{m}{({x}_{js}\times {b}_{s}-\bar{{x}_{j}\times b})}^{2})},$$

where *b* is a bit vector of size “m”. If sample e_s_ is in “J” then we set the s^th^ bit of *b*_*s*_ = 1, otherwise we set *b*_*s*_ = 0. The terms x_is_ and x_js_ represent s^th^ sample values for gene g_i_ and g_j_ respectively. Average expression values of g_i_ and g_j_ for samples in “J” are represented as $\overline{{x}_{i}\times b}$ and $\overline{{x}_{j}\times b}$ respectively. We considered a pair of genes g_i_ and g_j_ similar for a subset of samples “J” if $|r{(gi,gj)}_{J}| > \theta$, where *θ* is the correlation threshold and $|r{(gi,gj)}_{J}|\,\,$is the absolute value of correlation between g_i_ and g_j_ for the subset of samples “J”. For each gene g_i_ in a dataset “D”, CCS considers g_i_ as the base gene to start forming a bicluster for each g_i_.

### Search space sorting and base-gene selection

The gene with the lower variability over the samples (commonly known as housekeeping genes) are often considered as less significant for the corresponding biological conditions, hence the less important candidates for forming a bicluster. Prior to biclustering, the search space (input data matrix) was sorted by the standard deviations of the gene expression values. In an ordered data matrix the genes with the higher variability were placed before the other with the lower variability.

CCS algorithm selects ‘*base_number*’ of genes as base-gene from the sorted data matrix in the decreasing order of variability. Ordering of the search space ensured that a gene with higher variability considered for forming a bicluster and also for augmentation before the other with lower variability. The ‘*base_number*’ can be set between “1” to “n” (total number of genes). Here we set the *base_number* value equals 1,000 for the biological dataset to restrict the search time.

### Similarity pattern classes and sample selections

CCS algorithm considers three classes of gene expression pattern similarities: (i) up-regulated positive correlation: g_i_ and g_j_ are positively correlated and the expression values of g_i_ and g_j_ for the selected samples are higher than the arithmetic mean expression values over all smaples; (ii) down-regulated positive correlation: g_i_ and g_j_ are positively correlated and the expression values of g_i_ and g_j_ for the selected samples are lower than the arithmetic mean expression values over all smaples; (iii) negative correlation: g_i_ and g_j_ are negatively correlated over the selected samples and their expression values are at the opposite sides to their respective arithmetic mean expressions.

For each pair of genes g_i_ and g_j_ the sample sets “J_1_: for up-regulated positive correlation”, “J_2_: for down-regulated positive correlation” and “J_3_: for negative correlation” are selected from sample selection rules 1, 2 and 3 respectively. For each sample e_s_, s = 1:m, we defined the following three rules to determine J_1_, J_2_ and J_3_: **Rule 1**. IF $({x}_{is}-\overline{{x}_{i}}) > 0\,AND\,({x}_{js}-\overline{{x}_{j}}) > 0$ THEN include e_s_ to J_1_ [Expression values of g_i_ and g_j_ for the samples in J_1_ are higher than the arithmetic mean expre_s_sion values], **Rule 2**. IF $({x}_{is}-\overline{{x}_{i}}) < 0\,AND\,({x}_{js}-\overline{{x}_{j}}) < 0$ THEN include e_s_ to J_2_ [Expression values of g_i_ and g_j_ for the samples in J_2_ are lower than the arithmetic mean expression values], **Rule 3**. IF $({x}_{is}-\overline{{x}_{i}})\times ({x}_{js}-\overline{{x}_{j}}) < 0$ THEN include e_s_ to J_3_ [Expression values of g_i_ and g_j_ for the samples in J_3_ are opposite to their arithmetic mean expression values]. Here $\overline{{x}_{i}}$ and $\overline{{x}_{j}}$ are the mean expression value of gene g_i_ and g_j_ over all samples. The correlations between g_i_ and g_j_ for sample sets J_k=1:3_ are determined first by computing Pearson Correlation Coefficient ‘r’ and then by comparing against a threshold ‘θ’ which is denoted by |r(g_i_, g_j_)_Jk_| > θ in the algorithm.

### Defining biclusters as condition dependent correlation modules

CCS defines biclusters as condition dependent correlation modules where the genes in a bicluster are expected to show correlations only for the samples in that bicluster. CCS introduces a scoring function named BScore as defined in Equation (2). The BScore is designed to compare two sets of correlated gene pairs N and M. The correlations in set ‘N’ are computed over the samples that are included in a bicluster while the correlations in set ‘M’ are computed over the rest of the samples that are not included in the same bicluster. Thus, the BScore measures the degree to which the gene coexpression in a bicluster is condition-specific. In this work, we used a small BScore threshold (<0.01) to ensure the discovered biclusters are based on condition-specific coexpression.

2 $${\rm{BScore}}=\frac{|N{\cap }^{}M|}{|N{\cup }^{}M|}$$

### Algorithm

The algorithm at each iteration of step 2 starts with a new base-gene g_i_. In each iteration of step 5, g_i_ is paired with a gene g_j_ (i < j ≤ n). In step 6, the sample sets are selected for gene pair g_i_, g_j_. In step 8, the algorithm computes the absolute value of the correlation |r(g_i_, g_j_)_Jk_| for each sample set J_k=1:3_ (Equation (1)). If |r(g_i_, g_j_)_Jk_|$> \theta$ then the algorithm starts a bicluster with an initial gene set I_i_ = {g_i_, g_j_}. In step 10, augmentation of the gene set is performed by including a new gene g_p_ in I_i_. In step 16, ${\rm{BScore}}$(I_i_,J_i_) _i_s compared against the previous best ${\rm{BScore}}$(I,J) to update the sets I and J. The most condition dependent bicluster for each base gene g_i_ is selected in step 22. In step 26, all the overlapping biclusters are merged while keeping ${\rm{BScore}} < 0.01$

**Table:** 

**Input:** (i) A gene expression data set D = G × H, G = {g_1_, g_2_,…, g_n_} is a set of n genes, H = {e_1_, e_2_,…, e_m_} is a set of m samples for gene set G. (ii) A correlation threshold θ.
**Output:** A set of biclusters S = {bicluster(g_1_), bicluster(g_2_),…, bicluster(g_base_number_)}, base_number ≤ n.
1. S ← NULL
2. **for all** g_i_ $\in \,$G, i ≤ base_number **do**
3. I ← NULL
4. J ← NULL
**5**. **for all** g_j_ $\in \,$G, i < j ≤ n **do**
6. apply Rules_(1,2,3)_ on {g_i_, g_j_} for sample sets J_k(k=1,2,3)_
7. **for all** J_k_, k = 1:3 **do**
8. **if** |r(g_i_, g_j_)_Jk_| > θ **then**
9. I_i_ ← {g_i_, g_j_}
10. **for all** g_p_ not in I_i_ **do**
11. **if** |r(g_p_, g_q_)_Jk_| > θ, for all g_q_ $\in \,\,$I_i_ **then**
12. I_i_ ← I_i_ ∪ g_p_
13. **end if**
14. **end for**
15. **end if**
16. **If (**BScore(I_i_,J_i_) < BScore(I,J) < 0.01**) or**
**(**BScore(I_i_,J_i_) = BScore(I,J) < 0.01 **and** |I_i_| > |I|**) then**
17. I ← I_i_
18. J ← J_i_
19. **end if**
20. **end for**
21. **end for**
22. **if** I ≠ NULL and J ≠ NULL **then**
23. bicluster(g_i_) ← {I, J}
24. **end if**
25. **end for**
26. **for** each bicluster({I, J}) **do**
27. **for** each bicluster({K, L})$\ne \,$NULL **do**
28. **if** BScore(I ∪ K, J ∪ L) < 0.01 **and** I ∩ K ≠ NULL **then**
29. bicluster({I, J}) ← {I ∪ *K*, *J* ∪ *L*}
30. bicluster({K, L}) ← NULL
31. **end if**
32. **end for**
33. **end for**

for the final set of biclusters ‘S’.

The input parameter for our biclustering algorithm is the correlation threshold θ, which is the minimum absolute value of correlation between genes in a bicluster. Allocco *et al*. investigated the relation between co-regulation and coexpression and came up with a conclusion that for a correlation greater than 0.84 there is at least 50% chance of co-regulation^19^. Here we set θ = 0.8. If the number of biclusters is zero for θ = 0.8 then we decrease θ by 0.05 until we get a nonempty set of biclustering result or θ drops to 0.

### GPU computing for biclustering analysis of large datasets

GPU was initially introduced for rendering video graphics on display devices. The GPU designing company NVIDIA introduced their CUDA architecture in 2007 for general purpose computing on graphics processing unit (GPGPU computing). The single instruction set and multiple dataset architecture (SIMD) of GPU is particularly useful for executing a single instruction over thousands of core processors in a GPU card. CUDA is currently the most popular programming model for general purpose GPU computing. CUDA C is an extension of regular C that supports GPGPU computing. The parallel part is implemented as a CUDA C kernel function. Thousands of instances of a CUDA C kernel function run parallel on a group of GPU core processors. Each group of GPU cores is also known as a thread block. Each thread block is a virtual processor formed from one or more GPU core processors, registers, local memories, shared memory and global memory. CUDA C kernel functions are sent to the GPU for parallel execution, and sequential parts of the code run on the CPU.

We used GPGPU computing to reduce the execution time. In this work, we executed our CUDA C code for CCS on NVIDIA Tesla K20 (2,496 CUDA cores) and K80 GPU (4,992 CUDA cores) accelerator. For compilation of CUDA C code we installed CUDA 7.5 toolkit in our Linux workstation. Transferring big datasets between CPUs and GPUs is time consuming. To reduce the data transfer overhead we moved the entire process of finding new biclusters to GPU.

Step 2 of CCS executes “n” times when “*base_number*” is set at “n” for a dataset with “n” rows. For each execution the inner loop at step 5 executes a maximum “n-1” times. To achieve increased speed from parallel GPGPU computing, we removed the step 2 loop from CCS algorithm and implement steps 5 to 25 of CCS as a CUDA C kernel function.

### Synthetic datasets

Five synthetic datasets were generated using the BiBench-0.2 python library^8^. The synthetic datasets contains two types of biclustering: Constant biclusters and Shift-Scale biclusters. Constant biclusters are sub-matrices with a constant value and Shift-Scale biclusters are biclusters that are formed from both shifting and scaling a base row by random numbers. Table 1 describes the synthetic datasets for their types, dimensions and number of true biclusters.

**Table 1:** Summary of the synthetic datasets.

Dataset	Type	Rows	Columns	True biclusters
CNST.100.3	Constant	100	75	3
SS.150.4	Shift and Scale	150	100	4
SS.200.5	Constant	200	120	5
SS.200.6	Shift and Scale	200	120	6
SS.250.7	Shift and Scale	250	120	7

### Gene expression datasets

Five publicly available gene expression datasets GDS531, GDS589, GDS3603, GDS3966 and GDS4794 were downloaded from Gene Expression Omnibus (GEO). Genes with standard deviation less than 2.0 were removed. For genes appeared multiple times, only the row (probe set) with the highest standard deviation was kept. The dataset information is summarized in Table 2.

**Table 2:** Summary of the gene expression datasets and number of CCS biclusters obtained for θ = 0.8.

Dataset	Rows	Columns	Description	Number of CCS biclusters
GDS531	12625	173	Bone marrow plasma cell of multiple myeloma patients^39^	13
GDS589	8799	122	Peripheral and brain regions in rat strains^40^	20
GDS3603	12625	79	Advanced renal cancer Peripheral blood mononuclear cells^41^	14
GDS3966	22283	83	Melanoma clinical samples^42^	19
GDS4794	54675	65	Small cell lung cancer (SCLC) samples and normal samples^43^	19

### Evaluating biclustering results

We evaluated the results of the biclustering algorithms on synthetic datasets using Recovery and Relevance metrics^8, 20^. They compare the set of found biclusters ‘F’ against the expected biclusters ‘E’ (the true bicluster). Both for Recovery and Relevance the scores are ranging between ‘0’ and ‘1’. The highest score ‘1’ for ${\rm{Recovery}}$ denotes that all the expected biclusters are found. Similarly, the highest ‘1’ for ${\rm{Relevance}}$ denotes that all the found biclusters are expected.

To evaluate the results on the real gene expression datasets, we performed functional enrichment tests for each bicluster^1, 8, 13, 20^. We used gProfileR^21^ for selecting the enriched gene annotation terms. gProfilerR retrieves a comprehensive set of gene annotations from Gene Ontology (GO) terms, biological pathways, regulatory motifs in DNA, microRNA targets and protein complexes. From gProfiler search, all the annotation terms with a Benjamini-Hochberg FDR less than 0.01 were considered as enriched. From the biclustering results on all five gene expression datasets we computed the average number of enriched annotation terms. Higher average number of enriched terms indicates better functional grouping. We also evaluated the performance of biclustering algorithms from percentage of total biclusters that have one or more enriched annotation terms.

## Results and Discussions

We obtained the biclustering results from CCS and 13 other biclustering algorithms namely Cheng and Church Biclustering algorithm (CC)^22^, Iterative Signature Algorithm (ISA)^23^, Plaid^24^, Spectral^25^, Order Preserving Sub Matrix (OPSM)^26^, xMotifs^27^, SAMBA^28^, Bimax^20^, BicSPAM^29^, UniBic^30^, CPB^14^, BICLIC^15^ and BCCA^13^. We used BicAT^20^ software package for ISA, CC, OPSMs, xMotifs and Bimax. We also used ‘biclust’ R package (https://cran.r-project.org/web/packages/biclust/) for Plaid and Spectral. For UniBic, BicSPAM, BCCA, CPB and BICLIC the published versions of the software packages were used. SAMBA biclustering was performed using the Expander package^31^.

### Performance on synthetic data

**Figure 1:**
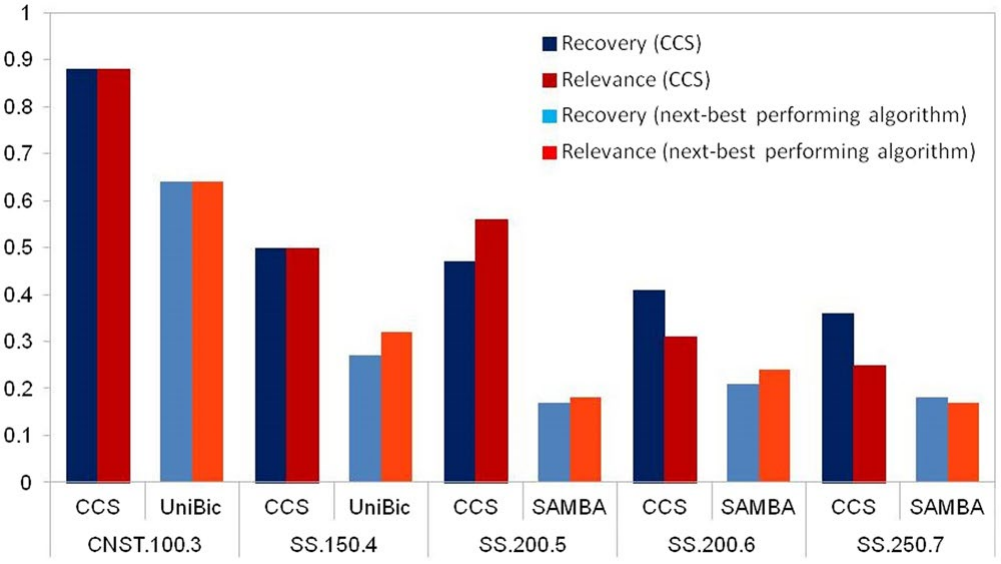
Recovery and Relevance scores on five synthetic datasets for CCS and the next-best performing algorithm.

Figure 1 shows the Recovery and Relevance scores on five synthetic datasets (Table 1) for CCS and the best among the other 13 algorithms for comparison. For all of the five synthetic datasets CCS significantly outperformed other algorithms for finding the expected biclusters (recovery) and the most relevant set of biclusters (relevance). For constant bicluster on a datasets of 100 rows and 100 columns, CCS obtained 0.88 score for both recovery and relevance. For larger size datasets and shift and scale biclusters, still the recovery and relevance scores are higher than the other algorithms which clearly demonstrate the accuracy of CCS for recovering the relevant biclusters irrespective of the number and pattern of the biclusters and size of the datasets.

### Performance on gene expression data

**Figure 2:**
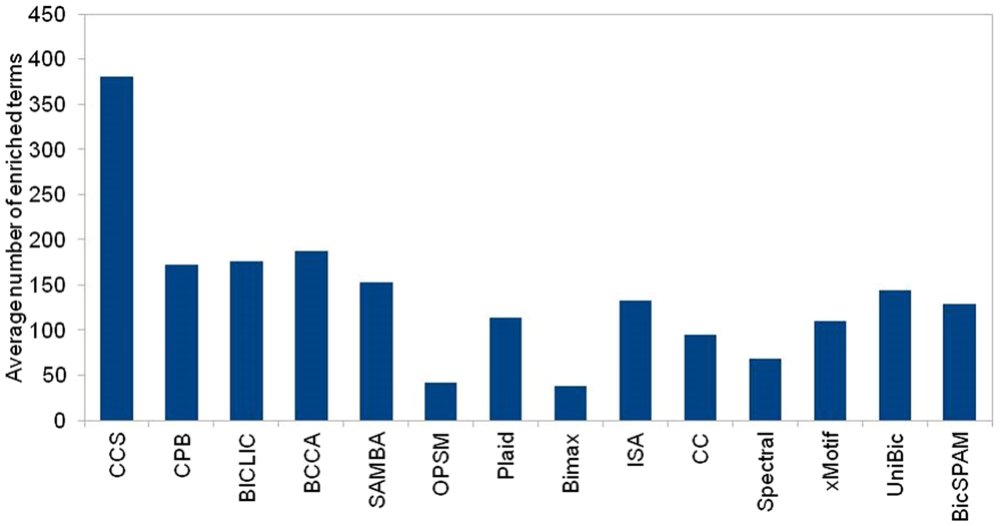
Average number of enriched terms on five gene expression datasets. All the enriched gene ontology terms with Benjamini-Hochberg FDR less than 0.01 were considered.

**Figure 3:**
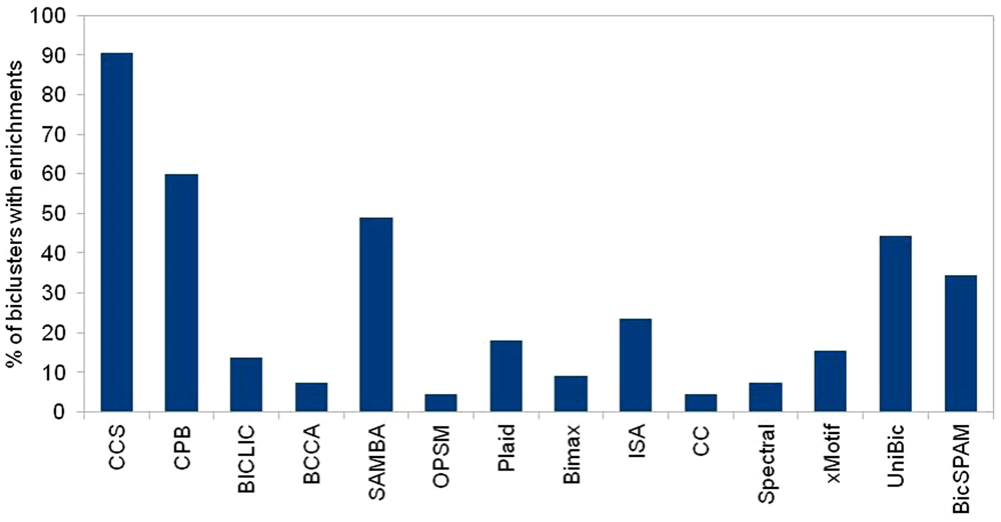
Percentage of bicluster from five gene expression datasets that have at list one enriched term. All the enriched gene ontology terms with Benjamini-Hochberg FDR less than 0.01 were considered.

CCS biclustering algorithm obtained total 25 biclusters from five gene expression datasets (Table 2). Figure 2 shows the average number of enriched terms from gProfiler enrichment analysis. Figure 2 shows there are more enriched terms per bicluster from CCS than the others. Figure 3 compares the biclustering algorithms for the percentage of biclusters with at least one enriched term. Again, CCS obtained the highest percentage of enriched biclusters (Figure 3).

### GPU accelerated biclustering

**Figure 4:**
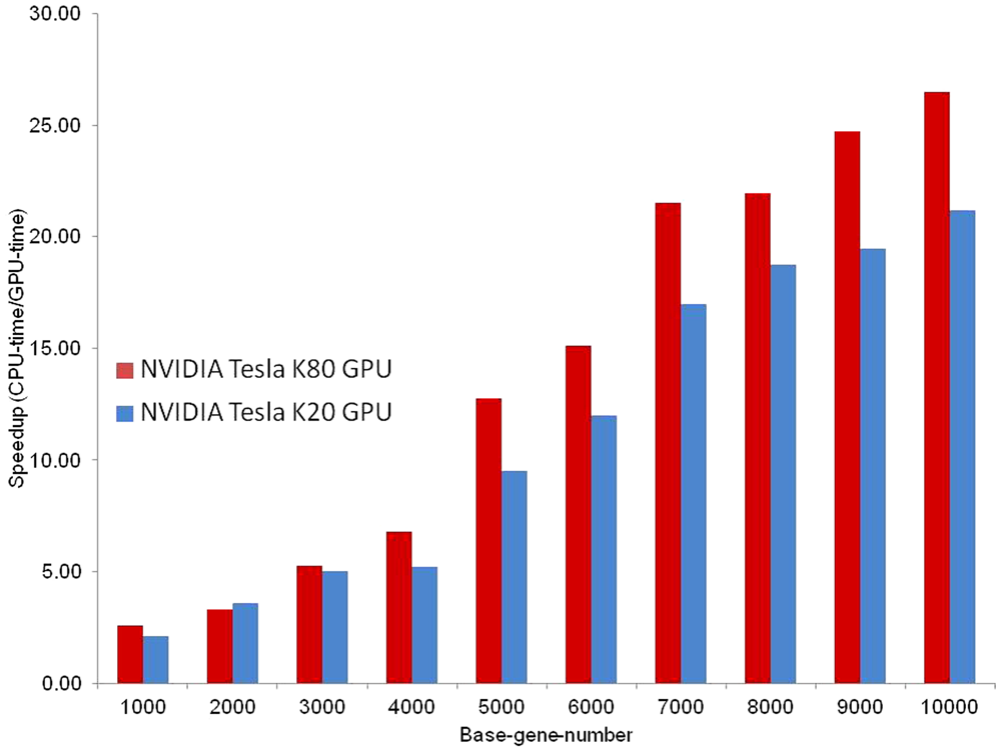
Comparison between the execution time for the CPU and GPU implementations of CCS on gene expression dataset GDS531. The “x” axis shows the number base genes for bicluster search. The “y” axis shows GPU speedup from CPU vs. GPU execution times.

The CPU version of the CCS algorithm is computationally expensive. If the ‘base_number’ is ‘n’ then step 5 executes ‘n’ times. Again for ‘n’ iterations of step 5 the augmentation task at step 10 executes ‘n’ times. Hence, the worst case time complexity of CCS is O(n^3^). The CUDA C implementation of CCS eliminates the outer loop and runs from step 5 to step 25 as CUDA C kernel function. This reduces the time complexity to O(n^2^). We compared the execution time of the CPU and GPU implementations of CCS. Figure 4 shows the speedup gained from CCS running on the NVIDIA Tesla K20 and K80 GPUs. For larger datasets the GPU-based CCS runs more than 20 times faster than the CPU-based CCS.

### CCS biclusters are equivalent to condition-dependent coexpression network modules

**Figure 5:**
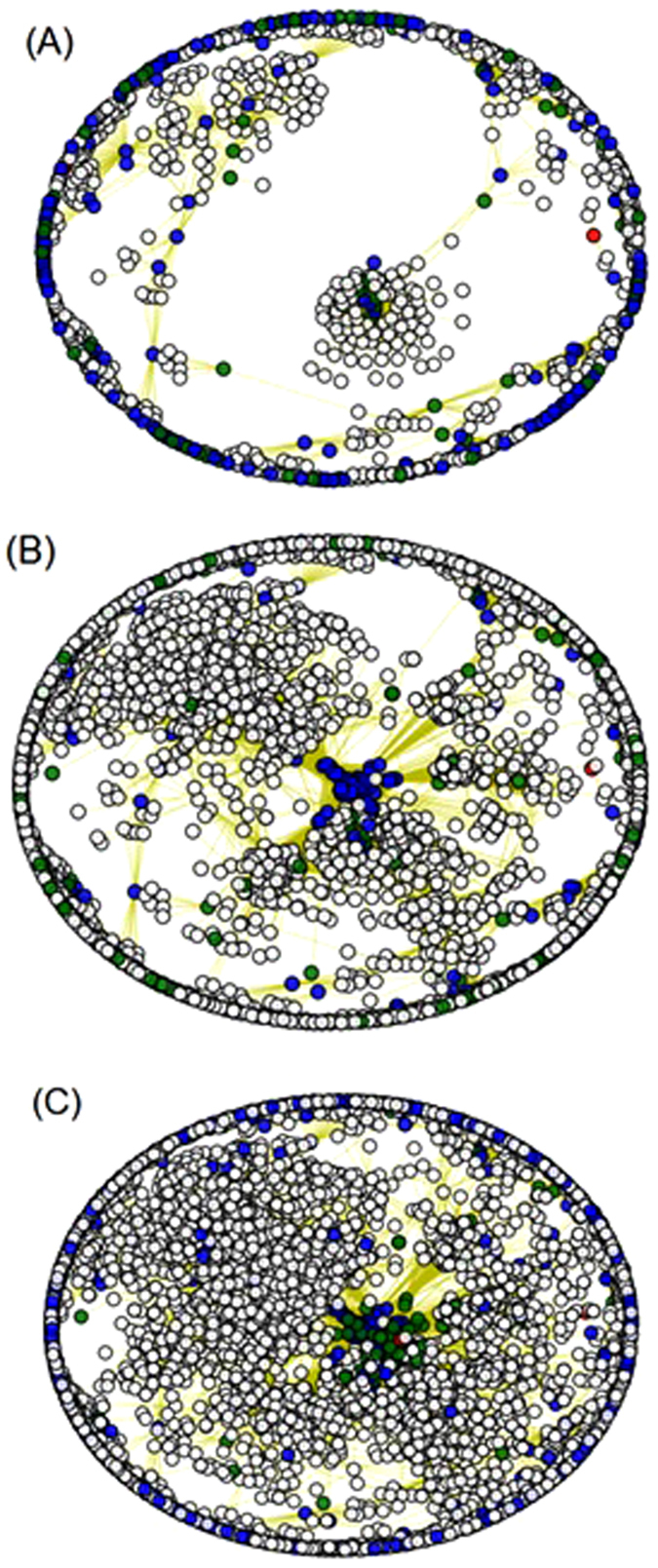
The coexpression network related to two CCS biclusters from GDS589. The blue, green and white nodes represent the genes from bicluster 1, bicluster 2 and the neighboring genes respectively. Red nodes are common genes between bicluster 1 and 2. Edges represent correlation >=0.8 or <=−0.8. (**A**) Coexpression network based on correlations over all the samples in GDS589. (**B**) Coexpression network based on correlations over the samples of bicluster 1 (**C**) Coexpression network based on correlations over the samples of bicluster 2.

A network module is usually defined as a highly connected sub-network. A module in coexpression network consists of a group of genes whose expression levels are highly correlated^32^. The correlative relations in a coexpression network often depends on conditions such as genotype, environment, treatment, cell type, disease state or developmental stage^33–38^. Therefore, coexpression network modules may also change with those conditions. There is an interesting relation between CCS biclusters and coexpression network modules. A CCS bicluster consists of genes whose expression levels are highly correlated under a set of conditions. Therefore, a CCS bicluster is equivalent to a condition-dependent module in a coexpression network. This has been illustrated here from two CCS-biclusters. We picked two CCS-biclusters from GDS589 dataset. The first bicluster (bicluster 1) includes 166 genes and 70 samples. The second bicluster (bicluster 2) includes 81 genes and 60 samples. We also identified all the neighboring genes of the biclusters. Neighboring genes are the genes that are not from a bicluster but they are correlated with at least one gene in that bicluster. Figure 5(A) shows the coexpression network of the genes in two biclusters and their neighboring genes. Because this network shows coexpression over all the samples, none of the biclusters are forming a network module. In contrast, when the coexpression network is constructed using correlations over the samples of bicluster 1, the genes of this bicluster form a network module, but the genes of bicluster 2 do not form a module (Figure 5(B)). Similarly, genes of bicluster 2 form a module when the coexpression network is based on correlations over samples of bicluster 2, but genes from bicluster 1 do not form a module in this case (Figure 5(C)).

## Conclusion

In this work we designed a novel algorithm, CCS, to discover the functionally coherent biclusters from large-scale gene expression datasets. The performance of CCS was compared with thirteen widely used biclustering algorithms. CCS consistently outperforms all the other algorithms in the comparison for obtaining true biclusters from synthetic datasets and discovering functionally enriched biclusters from the real gene expression datasets. Moreover, the CCS biclusters are equivalent to condition-dependent modules in coexpression networks. This important feature makes the CCS algorithm also useful for the study of the condition-dependent structural characteristics of the coexpression networks. The biclusters and network modules of co-regulated and co-functional genes discovered by the CCS algorithm may provide an important entry point for many other analyses such as gene set enrichment analysis, regulatory network inference and disease genes identification.

The recent development of fast and accurate data acquisitions and quantifications in genomics, transcriptomics and proteomics greatly increased the data volume that needs to be processed by clustering algorithms. To enable rapid discovery of high quality biclusters, we implemented CCS algorithm using CUDA C for parallel GPGPU computing. For large datasets, the GPU implementation of CCS achieved about 20 fold speedup compared to the sequential version of CCS. We expect that the GPU- accelerated biclustering will have important applications in the time-sensitive analysis of genomic medicine data.
